# Correction to ‘Pharmacological Interventions for Hereditary Transthyretin‐Related Amyloidosis With Polyneuropathy: Systematic Review and Network Meta‐Analysis’

**DOI:** 10.1111/ene.70696

**Published:** 2026-07-07

G. Duarte, T. Machado, F. Rodrigues, I. Conceição, D. Pareyson, J. da Costa, and on behalf of the Task Force of the EAN/PNS Joint Guideline for the Management of Neurological Manifestations in Hereditary Transthyretin Amyloidosis, “Pharmacological Interventions for Hereditary Transthyretin‐Related Amyloidosis With Polyneuropathy: Systematic Review and Network Meta‐Analysis,” *European Journal of Neurology* 33, no. 6 (2026): e70627. https://doi.org/10.1111/ene.70627.

In this article, two errors were identified.

In the list of authors, the fourth name was published incorrectly as Isabel Conceção. It should have been Isabel Conceição.

The concluding paragraph of the Discussion was published as:

In conclusion, gene‐silencing therapies, particularly the RNA interference agents patisiran and vutrisiran, demonstrated the most favourable efficacy profile across neuropathy impairment and quality‐of‐life outcomes. Given the above‐mentioned limitations, the present findings should be regarded as hypothesis‐generating rather than definitive comparative evidence. They represent the best available synthesis of the existing randomised evidence but cannot substitute direct head‐to‐head trials.

It should have been:

In conclusion, transthyretin gene‐silencing therapies as a class were more efficacious than stabilisers across neuropathy impairment and quality‐of‐life outcomes. Within the gene‐silencer class—patisiran, vutrisiran and eplontersen—the available evidence did not establish robust differences between individual agents: on the patient‐reported co‐primary outcome (Norfolk QoL‐DN) all three produced clinically meaningful improvements exceeding the minimal clinically important difference, with no statistically significant differences between them and overlapping rankings, while any apparent advantage of an individual RNA interference agent based on the mNIS+7 was derived from indirect, scale‐harmonised comparisons of very low to moderate certainty, with overlapping credible intervals and no head‐to‐head data. Given the above‐mentioned limitations, the present findings should be regarded as hypothesis‐generating rather than definitive comparative evidence. They represent the best available synthesis of the existing randomised evidence but cannot substitute for direct head‐to‐head trials, and should not be read as establishing the superiority of any single gene‐silencing agent over another.

These corrections refine the interpretation of the findings without affecting the conclusions; the underlying data, analyses, and results are unchanged.

The authors apologize for these errors.

